# A comparative biodistribution study of polymeric and lipid-based nanoparticles

**DOI:** 10.1007/s13346-022-01157-y

**Published:** 2022-04-15

**Authors:** Andreas K. O. Åslund, Rob J. Vandebriel, Fanny Caputo, Wim H. de Jong, Christiaan Delmaar, Astrid Hyldbakk, Emilie Rustique, Ruth Schmid, Sofie Snipstad, Isabelle Texier, Kai Vernstad, Sven Even F. Borgos

**Affiliations:** 1 Dept. Biotechnology and Nanomedicine, SINTEF Industry, Trondheim, Norway; 2 National Institute of Public Health and the Environment (RIVM), Bilthoven, Netherlands; 3 Université Grenoble Alpes, CEA LETI MINATEC Campus, Grenoble, France; 4 Department of Physics, Norwegian University of Science and Technology, Trondheim, Norway; 5 Cancer Clinic, St. Olavs Hospital, Trondheim, Norway

**Keywords:** Nanomedicine, Nanobiomaterial, Biodistribution, ADME, Poly(alkyl cyanoacrylate), Nanostructured lipid carrier

## Abstract

Biodistribution of nanoencapsulated bioactive compounds is primarily determined by the size, shape, chemical composition and surface properties of the encapsulating nanoparticle, and, thus, less dependent on the physicochemical properties of the active pharmaceutical ingredient encapsulated. In the current work, we aimed to investigate the impact of formulation type on biodistribution profile for two clinically relevant nanoformulations. We performed a comparative study of biodistribution in healthy rats at several dose levels and durations up to 14-day post-injection. The studied nanoformulations were nanostructured lipid carriers incorporating the fluorescent dye IR780-oleyl, and polymeric nanoparticles containing the anticancer agent cabazitaxel. The biodistribution was approximated by quantification of the cargo in blood and relevant organs. Several clear and systematic differences in biodistribution were observed, with the most pronounced being a much higher (more than 50-fold) measured concentration ratio between cabazitaxel in all organs vs. blood, as compared to IR780-oleyl. Normalized dose linearity largely showed opposite trends between the two compounds after injection. Cabazitaxel showed a higher brain accumulation than IR780-oleyl with increasing dose injected. Interestingly, cabazitaxel showed a notable and prolonged accumulation in lung tissue compared to other organs. The latter observations could warrant further studies towards a possible therapeutic indication within lung and conceivably brain cancer for nanoformulations of this highly antineoplastic compound, for which off-target toxicity is currently dose-limiting in the clinic.

**Graphical abstract:**

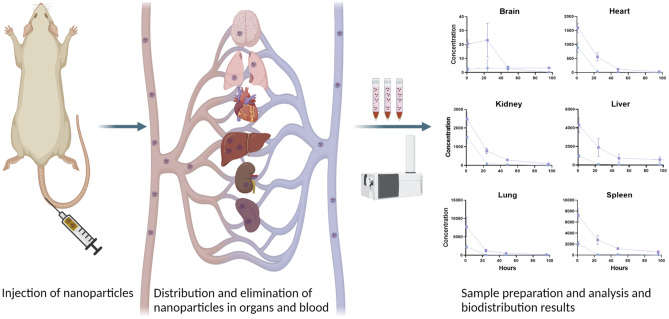

**Supplementary information:**

The online version contains supplementary material available at 10.1007/s13346-022-01157-y.

## Introduction

More than 50 nanomedicine formulations are already approved for therapeutic use in the clinic, and more than 400 clinical trials involving nanomedicines were active or recruiting upon assessment in 2020. These nanomedicines belong to a wide range of materials and formulation types, including liposomes; other lipid-based and polymeric micelles; and protein-based and inorganic nanoparticles [1]. Furthermore, nanomedicines have recently received unprecedented, global attention even in the lay population, as a consequence of their crucial role in the mRNA-based vaccines against Covid-19. In that case, lipid nanoparticles (LNPs) are used to encapsulate the active pharmaceutical ingredients (API) which are viral protein-encoding mRNA. The vaccines are injected locally and intramuscularly, and the primary function of the LNPs is to protect the mRNA from degradation before cellular uptake and endosomal escape into their cytoplasmic target compartment. Most nanocarriers currently used in the clinic, however, are delivered intravenously and will then be distributed systemically in the body through the blood circulation. Organs perfused by this circulation will retain and convert these nanoparticles to varying extent, defining their overall absorption, distribution, metabolism and excretion (ADME). A range of nanoparticle properties affect ADME, including their size, shape, surface charge and surface functionalities as reviewed by others [2, 3].

One major promise of nanomedicines has been the capacity to provide precise, targeted delivery of the active drug to the desired site of pharmaceutical effect (e.g. the tumour), thus maximizing the therapeutic efficacy whereas at the same time reducing off-target toxicities. The propensity to accumulate in the liver constitutes an inherent ‘passive targeting’ of the LNP-based Onpattro^®^ formulation of siRNA that acts against hereditary transthyretin-mediated amyloidosis, and that has the liver as its target organ. Extending from this, a very interesting, recent example of organ-selective targeting by alteration of the lipid composition in LNPs was presented by Cheng et al. [4]. The ‘holy grail’ of achieving active, cell-specific targeting by nanoparticle surface ligand attachment is still considered challenging, although progress has been made [5]. Beyond targeting to specific organs, a prolonged circulation, and thus availability of the API in the blood stream, by particle surface modification with polyethylene glycol (PEGylation) is frequently used to enhance treatment effect. This is achieved by keeping the accessible API above minimum therapeutic concentrations but below unacceptable toxicity without the need for excessively frequent (re-)administration, as is the case e.g. for the clinically used paclitaxel formulation Abraxane^®^. The first nanomedicine introduced to the clinic in 1995, Doxil^®^ (liposomal doxorubicin), has as one of its main therapeutic benefits to the reduction of off-target cardiotoxicity as compared to free doxorubicin.

The biodistribution, clearance and systemic fate of clinically relevant nanomaterials have been thoroughly reviewed by Bourquin et al. [6]. It is clear that although the single most determining factor for biodistribution of injectable nanomaterials is the presence of PEG surface groups to avoid clearance by the reticuloendothelial system (RES), there are still significant differences between e.g. lipidic, polymeric and inorganic nanoparticles in terms of biodistribution. Furthermore, as emphasized by the authors, is it important to keep in mind that for controlled-release formulations, a distinction should be made between the encapsulated and the released API. Only the latter is able to exert its pharmaceutical activity.

The EU H2020 project REFINE aims to support the science-based development and optimization of a regulatory framework for nanobiomaterials, encompassing medicinal products and the potentially released nanosized components and/or wear of medical devices. In a previous analysis performed under REFINE [7], ADME, including biodistribution, was identified as one of five main areas where methodological gaps exist in the preclinical safety assessment of nanobiomaterials, motivating the current study. Two model materials are used in REFINE, representing the two major organic formulation classes: lipids and polymers.

The first lipid-based nanomedicine, Doxil®, as well as the majority of currently used nanomedicines in the clinic, is liposomes, composed of an aqueous core surrounded by a phospholipid bilayer that may consist of a single or multilamellar layer. Whereas the aqueous liposome core is well suited to contain and deliver relatively hydrophilic drugs, the capacity to incorporate hydrophobic drugs is limited to the lipid bilayer. Nanoparticles entirely composed of lipids will have a higher hydrophobic volume fraction, and thus conceivably a higher hydrophobic drug loading capacity [8, 9]. Furthermore, they can be made non-toxic and biodegradable [10], being mainly composed of Food and Drug Administration (FDA)- and European Medicines Agency (EMA)-approved ingredients: their lipid composition remains alike to lipophilic physiological molecules but adapted to fit the encapsulated active molecule. Nanostructured lipid carriers (NLC) constitute a recent generation of such lipid-based delivery systems. Contrary to solid lipid nanoparticles (SLN) and lipid nano-emulsions (LNE) which present a lipid core that is in a solid or liquid state at room temperature, respectively, the NLC core is composed of a blend of liquid and solid lipids, potentially able to entrap higher payloads of active molecules, while better controlling their release from the blend of lipids [11]. Over the last two decades, the interest of NLC for delivery applications has steadily increased [12, 13], largely due to their safe bioassimilable ingredients, their up-scalable fabrication process [14] and their long-term colloidal stability [8, 11]. In the REFINE project, we have used a NLC that has demonstrated great promise for imaging purposes [15, 16] and the delivery of active pharmaceutical ingredients [17–19]. This NLC platform was loaded with IR780-oleyl, a near-infrared (NIR) dye tailored for its efficient encapsulation in the particle lipid core, yielding LipImage^™^ 815, a nano-imaging agent for in vivo near-infrared (NIR) fluorescence imaging [20]. These nanoparticles are based on ingredients approved for human use, have high biocompatibility, are produced free of toxic solvents and have undergone production scale-up [16]. They showed low cytotoxicity and a prolonged plasma circulation in mice and larger animals [15, 20, 21].

Polymers constitute another broad and major class of nanobiomaterials [22–25], and include both dendrimers [26] and other polymer-based prodrugs [27, 28]. Polymer-based systems exhibit a range of distinct advantages, such as high and controllable structural and chemical stability, broad spectrum of base material composition and, crucially, a very wide range of chemical functionalities, both for control of physicochemical and stability parameters and for the attachment or complexation of various drugs. The first polymeric nanoformulation, based on poly(lactic-co-glycolic acid) (PLGA), was approved for clinical use by the FDA already in 1989. In the REFINE project, we are formulating and studying another highly promising polymeric platform for drug delivery, based on the biodegradable polymer class poly(alkyl cyanoacrylate) (PACA) [29], which has been used in the clinic as adhesives for decades. PACA nanoparticles can be prepared with high drug loading and limited burst release [30]. Specifically, a miniemulsion polymerization process can be used to nanoformulate poly(2-ethylbutyl cyanoacrylate) (PEBCA), incorporating the semisynthetic taxane cabazitaxel (Cbz). Cbz is a second-generation taxane, and compared to docetaxel and paclitaxel, it has low affinity for P-glycoprotein efflux pumps, making it suitable for tumours that exhibit resistance by increased expression of P-glycoprotein [30]. Cbz is currently in use for second-line treatment of castration-resistant prostate cancer under the trade name Jevtana^®^. However, Cbz induces severe off-target toxicity including neutropenia, which severely limits its dose and wider use towards other cancers. Consequently, it is of interest to encapsulate Cbz in order to reduce acute toxic events and increase tumour targeting. Previous in vivo experiments in animal models have pointed to very promising therapeutic effects [31–34].

In this work, both lipid and polymer nanoformulations were use as model nanoscale drug delivery systems to evaluate their ADME profiles and discuss and compare the findings of their biodistribution as input in an in silico biodistribution model (published separately) and further as examples for regulatory testing of these types of nanomedicinal products.

## Materials and methods

### LipImage synthesis and characterization

Batches of LipImage^™^ 815 (henceforth ‘LipImage’) were prepared by high-pressure homogenization (HPH). The lipid phase comprised 19.125 g of soybean oil, 6.375 g of Suppocire^™^ NB, 4.875 g of lecithin and 150 mg of IR780-oleyl (molar mass: 986.29 g/mol), which was synthetized as previously described [20]. The aqueous phase comprised 25.875 g of Myrj^™^ S40 and 110 mL NaCl 154 mM. The mixture of lipid and aqueous phases was pre-emulsified using a mechanical disperser (Ultra-T25 Digital Turrax, IKA) operated at 15,000 rpm for 5 min. The emulsion was then processed with a high-pressure homogenizer (Panda Plus 2000, GEA Niro Soavi, Italy) operated for 16 cycles with a total pressure of 1250 bars, the pressure of the second stage chamber and the cooling system being set at 50 bars and 30 °C, respectively. Two hundred-gram batches of particles were then purified by 5 µm filtration followed by tangential flow filtration (Labscale TFF system, Millipore) against NaCl 154 mM through a Pellicon XL Biomax^™^ cassette (Merck) operated at a trans-membrane pressure of 14 psi at a flow rate of 2 mL/min. The nanoparticle dispersion was adjusted to a concentration of 100 mg/mL and filtered through a 0.22 μm Millipore membrane for sterilization before storage and use.

Dynamic light scattering (DLS) was used to determine the particle hydrodynamic diameter and zeta potential (Zeta Sizer Nano ZS, Malvern Instrument, Orsay, France). Particle dispersions were diluted to 2 mg/mL of lipids in 0.22-µm filtered 0.1 X PBS and transferred in Zeta Sizer Nano cells (Malvern Instrument) before each measurement, performed in triplicate. Results (Z-average diameter, dispersity index, zeta potential) were expressed as mean and standard deviation of three independent measurements performed at 25 °C. The encapsulation efficiency and payload of IR780-oleyl dye in the LipImage were determined by high-performance liquid chromatography (HPLC WATERS Alliance 2695/Fluorescence 2475 detector) and compared with a calibration curve established from the reference fluorophore IR780-oleyl alone, as previously described [35].

### PACA (PEBCA) nanoparticle synthesis and characterization

PACA nanoparticles (NPs), specifically based on ethylbutyl cyanoacrylate monomer and henceforth referred as PEBCA, were synthesized under aseptic conditions by miniemulsion polymerization. Prior to synthesis, all solutions were sterile filtered, and all equipment was autoclaved. An oil phase consisting of ethylbutyl cyanoacrylate (EBCA) (Cuantum Medical Cosmetics) containing 2 wt% Miglyol 812 N, 12 wt% cabazitaxel (BioChemPartner) and 2 wt% vanillin was used.

The oil phase was added to an aqueous phase consisting of 0.1 M HCl containing the two PEG-containing stabilizers (Brij^®^L23 and Kolliphor^®^HS15, both Sigma-Aldrich, 5 wt% of each). The water and oil phases were mixed and immediately sonicated for 3 min on ice (6 × 30 s intervals, 60% amplitude, Branson Ultrasonics Digital Sonifier). The solution was rotated (15 rpm) at room temperature overnight. The pH was then adjusted to 5.0 to allow further polymerization for 5 h at room temperature. The dispersions were dialyzed (Spectra/Por dialysis membrane MWCO 100.000 Da) against 1 mM HCl to remove unreacted PEGylated compounds. The size (z-average), polydispersity index (PDI) and the zeta potential of the NPs in phosphate buffer (10 mM, pH 7.0) were measured by dynamic light scattering (DLS) and laser Doppler micro-electrophoresis using a Zetasizer Nano ZS (Malvern Instruments). The dry weight of the NPs was calculated by drying 1 mL triplicates of the nanoparticle suspension and weighing the samples before and after drying.

To calculate the amount of encapsulated drug, the drug was extracted from the particles by dissolving them in acetone and quantified by liquid chromatography coupled to mass spectrometry (LC–MS/MS) (see below).

### LipImage animal experiments

Male Wistar rats, 6–8 weeks of age, were obtained from Envigo (Horst, the Netherlands) and housed in the animal facility of the Animal Research Centre (Bilthoven, the Netherlands). Animals were bred under SPF conditions and barrier maintained during the experiment. Drinking water and conventional feed were provided ad libitum. Husbandry conditions were maintained according to all applicable provisions of the national laws, Experiments on Animals Decree and Experiments on Animals Act. The experiment was approved by an independent ethical committee prior to the study.

LipImage, with an estimated lipid concentration of 96 mg/mL and IR780-oleyl concentration of 230 µg/mL, was used either undiluted, diluted to 33%, or diluted to 10%. The diluent was saline (0.9% NaCl) + 5% ethanol. The injection volume was 500 µL per 250-g body weight (weight established on the day before treatment), resulting in dose levels of 192, 64 and 21.3 mg/kg. The solutions remained clear and no sediments or agglomerates/aggregates were visually noted. Intravenous injection was done under isoflurane anaesthesia (Baxter). Per dose group, the animals were injected on the same day of the week, with weekly intervals between the dose groups in the order high – middle – low dose.

The number of animals per dose and time point was (*N*) = 4. At 15 min, 30 min, 60 min, 4 h and 24 h after injection, 1 mL of blood was collected through a venous puncture under isoflurane anaesthesia in MiniCollect K3-EDTA tubes (Greiner). The resulting plasma was snap frozen in liquid nitrogen. At 1 h, 1 day, 2 days, 4 days and 14 days after injection, the animals were euthanized by exsanguination from the heart puncture under ketamine/xylazine anaesthesia. Blood was collected in Vacuette K3-EDTA tubes (Greiner) and the resulting plasma was snap frozen in liquid nitrogen. The following organs were collected: brain, heart, kidney, liver, lung, spleen, testis and thymus. Organs were weighed, cut in 2 symmetric halves (except testes) and snap frozen in liquid nitrogen. Plasma and organs were stored at − 80 $^\circ{\rm C}$ and shipped on dry ice for analysis.

### PEBCA animal experiments

Male Wistar rats were purchased at 8 weeks of age from Janvier Labs (France). They were housed in groups of four in individually ventilated cages in a specific pathogen-free environment at 22–23 °C and 50–60% relative humidity, on a 12-h light/dark cycle and with 70 air changes per hour and free access to food and sterile water. They were fed RM1 expanded pellets (Special Diets Services, Essex, UK), and the cages were enriched with housing, nesting material and gnaw sticks. All institutional and national guidelines for the care and use of laboratory animals were followed. Experiments were performed in Norway.

During all experiments, the animals were anaesthetized by inhalation of 2–3% isoflurane (Baxter, Deerfield, IL, USA). Anaesthesia was maintained with isoflurane in 0.4 L/min O_2_ and 0.6 L/min N_2_O.

To perform the pharmacokinetics and biodistribution study, the animals were separated into 2 groups according to dose:

1. Low dose PEBCA-Cbz, 0.5 mg/kg Cbz (4.4 mg/kg NPs)
2. High dose PEBCA-Cbz, 3.5 mg/kg Cbz (30.5 mg/kg NPs)

Details about the treatment groups are available in Table 1. Before injection, the particles were diluted in 0.9% saline to a concentration that corresponded to an injection volume of 2 mL/kg. In addition to the treated animals, 6 sham-treated (injected with 0.9% saline 2 mL/kg) animals were used. The sham-treated animals were euthanized at 2, 4 and 14 days, two at each time point. Animals were injected intravenously once.

**Table 1:** NP dose injected in the different groups and different NPs

	Low dose		Medium dose		High dose	
	NP (mg/kg)	Load^1^ (mg/kg)	NP (mg/kg)	Load (mg/kg)	NP (mg/kg)	Load (mg/kg)
LipImage	21.3	0,051	64	0.15	192	0.46
PEBCA	4.4	0.5	-	-	30.4	3.5

^1^Load is either Cbz or IR780-Oleyl

Blood samples on time points 1–30 min and 4 h (Table 2) were performed on conscious animals, and all other blood samples were collected during exsanguination. The leg of the animal was shaved to expose the saphenous vein. Petrolatum was applied to the shaved area and the saphenous vein was punctured using a 19G needle. Into tubes containing blood clotting activator, 300–500 µL blood was collected (Microvette 500 Serum, Sarstedt AG & Co. KG, Germany). The tubes were turned 3–4 times, left for 60 min in upright position at room temperature to allow clot formation before centrifugation at 10 000 rcf for 5 min. The serum was immediately transferred to new tubes and stored at − 80 °C until LC–MS/MS analysis.

**Table 2:** Detailed information on sampling points and number of animals in the PEBCA experiment

**Euthanasia**	**Blood sampling**	**# animals low dose**	**# animals high dose**
1 h	1 h	4	4
1 day	3 min, 4 h, 1 day	4	4
2 days	7 min, 2 days	4	4
4 days	1 min, 15 min, 4 days	4	4
14 days	30 min, 14 days	4	4

**Table 3:** Procedure for homogenization

**Organ**	**Speed (on Ultra-Turrax)**	**Time (min)**
Brain	2	2
Heart	Gradual increase up to 6	4
Kidney	6	2
Testicles	9	2
Spleen	9	2
Thymus	9	2
Lung	9	4
Liver	9	2

**Table 4:** Maximal concentrations (*C*_max_) measured in tissues (ng/g) and serum (ng/mL), and sampling time (hours, minutes) at which this concentration was measured (*T*_max_). Concentrations are measured for Cbz after PEBCA injections and for IR780-oleyl after LipImage injections. Asterisk (*) indicates first that the sampling time is the first in the sampling series. Each data point shows the average of *n* = 4 animals

	**Brain**		**Heart**		**Kidney**		**Liver**		**Lung**		**Spleen**		**Serum**	
	*C*_max_ (ng/g)	*T*_max_ (h)	*C*_max_ (ng/g)	*T*_max_ (h)	*C*_max_ (ng/g)	*T*_max_ (h)	*C*_max_ (ng/g)	*T*_max_ (h)	*C*_max_ (ng/g)	*T*_max_ (h)	*C*_max_ (ng/g)	*T*_max_ (h)	*C*_max_ (ng/mL)	*T*_max_ (min)
**PEBCA low**	2.89	24	868	1*	1424	1*	205	1*	2235	1*	2072	1*	2198	1*
**PEBCA high**	22.4	24	1569	1*	1766	1*	912	1*	7593	1*	7083	1*	23,290	1*
**LipImage low**	2.08	1*	28.6	1*	24.2	1*	109	24	68.8	1*	33.1	48	1121	15*
**LipImage medium**	3.24	1*	84.4	1*	70.4	1*	285	24	188	1*	153	96	3007	15*
**LipImage high**	10.6	1*	178	1*	110	1*	945	24	515	1*	432	1*	6183	30

### Analyte extraction

For tissue distribution of LipImage^™^ 815 and PEBCA, IR780-oleyl and cabazitaxel, respectively, levels in organs were determined at the indicated time points. A solution of ethylene diamine tetra-acetate (EDTA, 5.1 mM in deionized water with 5% acetone) and a solution of 250 mM phenylmethylsulphonyl fluoride (PMSF) in absolute ethanol were prepared. The organ was transferred to a homogenization tube (DT-50 for liver and DT-20 for other organs; IKA-Werke GmbH & Co. KG). To DT-50 tubes, 20 mL of the EDTA solution and 200 µL of the PMSF solution were added. To DT-20 tubes, 15 mL of EDTA solution and 150 µl of PMSF solution were added. The organs were homogenized on an Ultra-Turrax tube drive according to Table 3. Four millimetres of the homogenate was transferred to a centrifuge tube containing two glass beads (5 mm) and lyophilized. To each sample, 0.5 or 2 ml acetone (smaller organs and liver, respectively) was added, and the samples were mixed on an automatic paint mixer (Jotun AS) for 12 min. For IR780-oleyl extraction, 0.1% Miglyol 812 N was added to the acetone. After centrifugation at 3700 rcf for 5 min, 100 µL of the supernatant was collected and used for further LC–MS/MS analysis.

Serum and plasma samples were diluted 1 + 9 in acetone/acetone-added Miglyol before quantification by LC–MS/MS.

### LC–MS/MS quantification of IR780-oleyl

IR780-oleyl was quantified by LC–MS/MS using an Agilent 1290 HPLC system coupled to an Agilent 6495 triple quadrupole mass spectrometer (Agilent Technologies, Santa Clara, CA, USA). An Ascentis Express C8 column (75 × 2.1 mm, 2.7 μm particles size, Sigma) with a 5 × 2.1 mm guard column of the same material was used for chromatographic separation. Mobile phase A was 10 mM ammonium acetate/0.1% formic acid in water and mobile phase B was 10 mM ammonium acetate/0.1% formic acid in a 5:2 (v/v) mix of acetonitrile and 2-propanol. The flow rate was 0.4 mL/min, and a chromatographic gradient was applied as follows: linear increase from 40 to 95% B over 7 min, 95% B for 2 min, linear increase to 100% over 2 min, 100% B for 3 min, before 1 min column re-equilibration (total run time: 15 min). Injection volume was 2.5 μL. The MS was operated in positive electrospray ionization (ESI) mode (Agilent Jetstream), using the m/z 858.4 → 521.1 transition for IR780-oleyl quantification.

The IR780-oleyl standard was the same as used for nanoparticle synthesis (see above). Standard solutions were prepared by dilution in acetone-added 1 mg/mL Miglyol 812 N (Cremer Oleo GmbH & Co. KG, Hamburg, Germany) to limit IR780-oleyl adhesion to vial walls and pipet tips. Standard curves ranging from 0.1 to 1000 ng/mL were fitted to a quadratic regression model using weighting factor 1/x.

### LC–MS/MS quantification of Cbz and metabolites

Cbz was quantified by LC–MS/MS, using the same instrumentation and chromatographic column as described for IR-780-oleyl quantification above. A binary gradient of mobile phase A (25 mM formic acid in water) and mobile phase B (methanol) was used as follows: 55% B for 1.5 min, linear increase to 70% B over 1 min, 70% B for 0.2 min, linear increase to 75% B over 0.5 min, 75% B for 0.5 min, 90% B washout step for 0.6 min and column re-equilibration at 55% B for 0.9 min (total run time: 5.2 min). The flow rate was constant at 0.5 mL/min and the injection volume was 1 µL. The MS was operated in positive electrospray ionization (ESI) mode (Agilent Jetstream). For quantitative analysis, the following multiple reaction monitoring (MRM) transitions were used for Cbz, 7-methyl-docetaxel, 10-methyl-docetaxel, docetaxel and 7-epi-docetaxel, respectively: m/z 858.3 → 577.2, 844.0 → 318.1, 844.0 → 563.0, 830.0 → 304.1 and 830.0 → 549.1. Similarly, Cbz-d6 and docetaxel-d5 internal standards were monitored at m/z 864.4 → 583.2 and 835.5 → 554.3, respectively.

The unlabelled Cbz standard was the same as used for nanoparticle synthesis (see above). 7-Methyl-docetaxel was purchased from SynZeal Research (Moraiya, India, catalogue number SZ-D031010), 10-methyl-docetaxel from Eos Med Chem (Jinan, China, custom synthesis), docetaxel from Sigma (Fluka, product number 01885) and 7-epi-docetaxel from Santa Cruz Biotechnology (Dallas, USA, product number SC-210610). Standard curves ranging from 0.1 to 1000 ng/mL were fitted to a quadratic regression model using weighting factor 1/ × for all analytes.

The Cbz-d6 and docetaxel-d5 internal standards were purchased from Toronto Research Chemicals (Toronto, Canada, catalogue numbers C046502 and D494423, respectively). A mix of the two internal standards was added to all standards and samples to yield a final concentration of 50 ng/mL.

All graphs were plotted using GraphPad Prism 9 and error par shows ± standard deviation.

## Results

### Nanoparticle characteristics

LipImage particle diameter was found by DLS to be 52.2 nm with a polydispersity of 0.102. The zeta potential was 0 mV, i.e. neutral. Particle dry weight in the dispersion was 95 mg/mL, and the IR780-oleyl concentration was 239.5 µM. For PEBCA, particle diameter was found to be 121.8 nm with polydispersity 0.14. Zeta potential was − 5.5 mV. Particle dry weight in the stock dispersion was 107 mg/mL, with Cbz loading 14.7 mM.

### Treatment of animals

All animals were monitored throughout the experiment time and exhibited no signs of reduced activity, stress or weight loss due to the treatments.

### Sample preparation optimization

Liquid chromatography coupled to tandem mass spectrometry (LC–MS/MS) was chosen for the accurate quantification of IR780-oleyl, Cbz and the metabolites docetaxel (Doc) and 10-methyl-docetaxel (10-m-Doc). These are all highly hydrophobic analytes, and although extraction and handling with high percentage of organic solvent is usually sufficient to ensure solubility, a particular challenge was observed for IR780-oleyl. In the low concentration range, almost complete loss of signal occurred despite extensive extraction optimization. Finally, it was found that adhesion of the IR780-oleyl was extremely strong to all glass and plastic disposables tested. A strategy was devised for competitive saturation of the surface binding sites where 1 mg/mL Miglyol 812 N was added to all solvents used. This permitted robust and reproducible detection down to 0.1 ng/mL IR780-oleyl injected.

### Serum concentrations of nanobiomaterial payloads show different time profiles

**Fig. 1:**
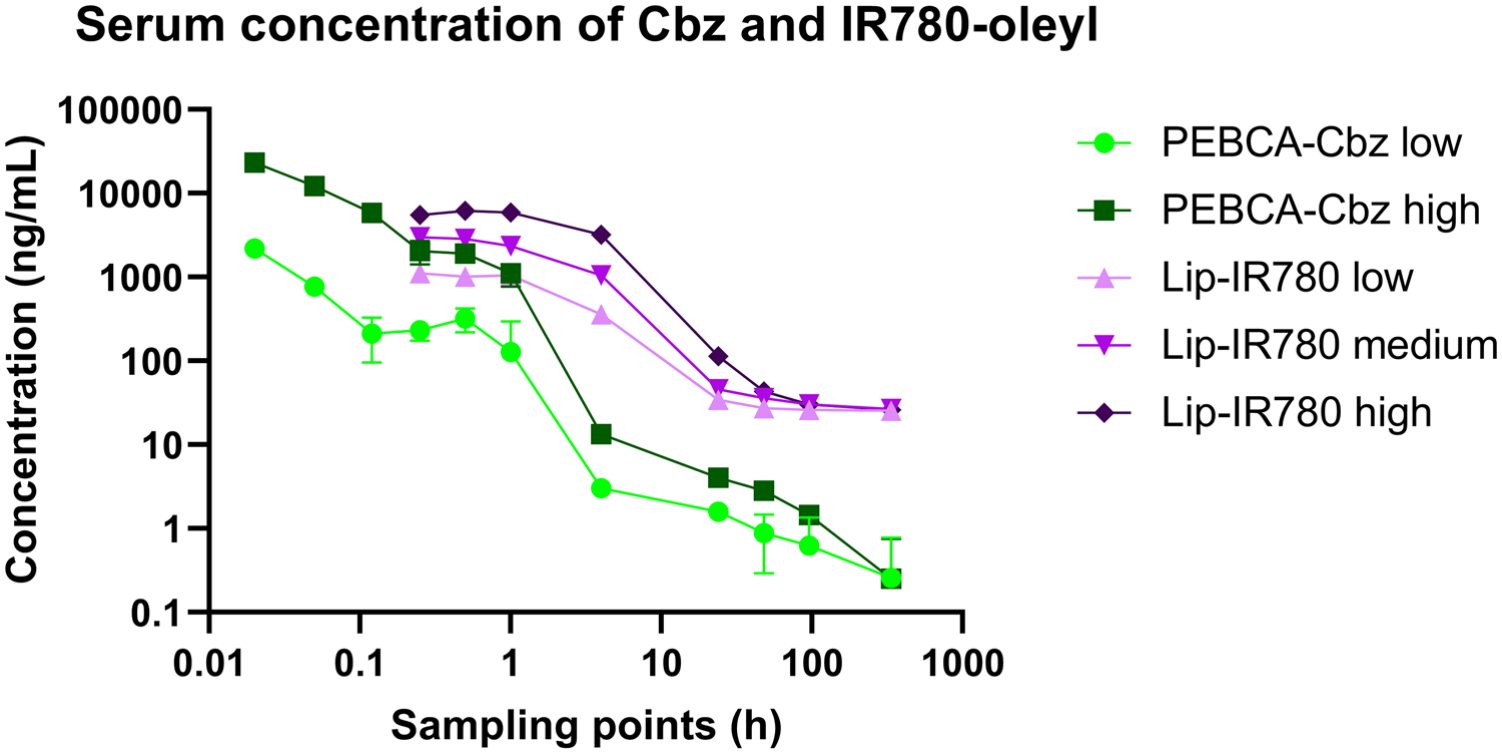
Measured concentrations of Cbz (from PEBCA) and IR780-oleyl (from LipImage) in serum (ng/mL) over the experiment duration, for all doses injected. Note that both axes are logarithmic. Each data point is the average of *n* = 4 animals

Large differences were seen in concentration profiles over time for IR780-oleyl and Cbz in serum after administration of the LipImage and PEBCA formulations (Figs. 1 and2). Scaling all dose regimes to the first common blood sampling time, i.e. 15 min, and plotting the data on logarithmic scales visualize the relevant trends. After injection, the serum kinetics of the two compounds differ in that the almost 100-fold decrease in concentration of Cbz takes place within the first 4 h, whereas IR780-oleyl lags this trend by more than 20 h. Interestingly, the total fold reduction in serum concentration over the period from the 15-min sampling point to the last point at 14 days is markedly higher for Cbz than for IR780-oleyl, as the latter seems to converge asymptotically towards a residual concentration of approx. 25 ng/mL. This residual concentration is reinforced in the bar graph, where the steepest increase in AUC occurs at later time points for IR780-oleyl as compared to Cbz.

**Fig. 2:**
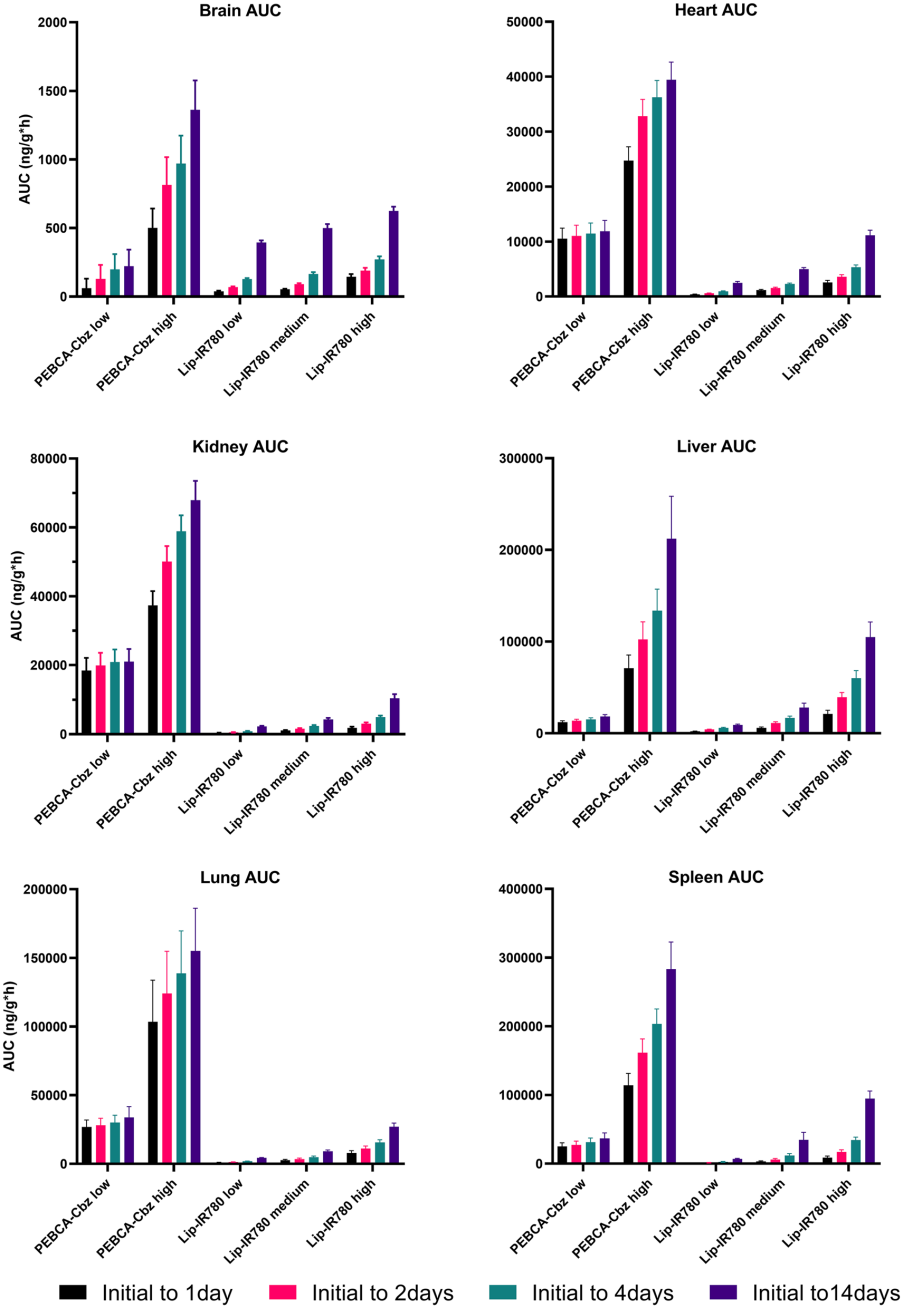

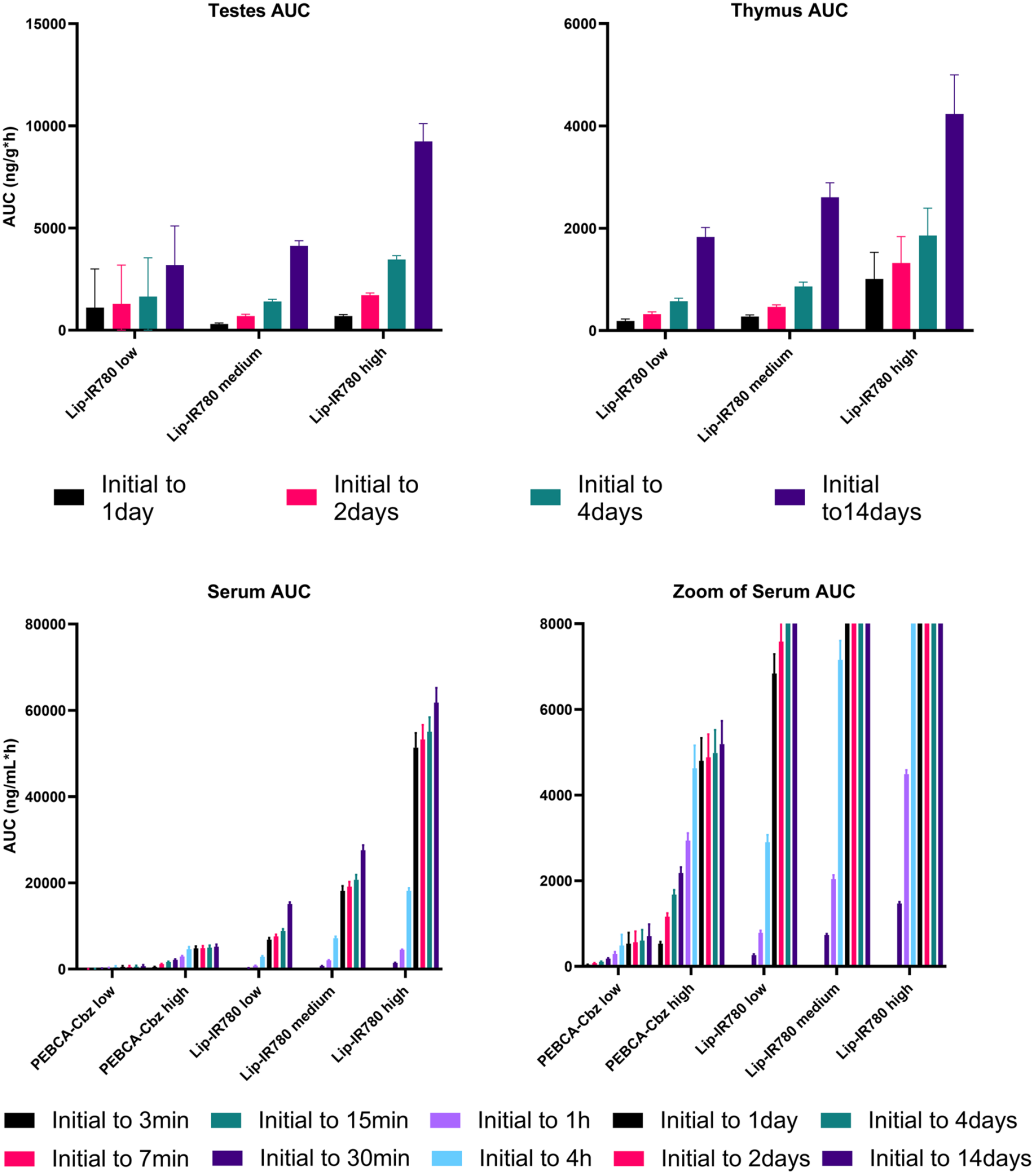
Area under the curve (AUC, in ng/g tissue or ng/mL serum * hour) for all organs and serum, all dose regimes. AUC is calculated for Cbz in PEBCA-injected animals and for IR780-oleyl in LipImage-injected animals. AUC is shown as an accumulation over time from the first sampling time (‘initial’; 1 h for organs, 1 min for serum in PEBCA injections, 15 min for serum in LipImage injections). Testes and thymus were not collected for PEBCA injections. Each data point shows the average and standard deviation of *n* = 4 animals

### Organ biodistribution of nanobiomaterial payloads is determined by formulation type

Figure 2 and Table 4 summarize the concentrations of payloads (Cbz and IR780-oleyl) found in all organs, for both formulations and all doses. Results are presented as accumulated area under the curve (AUC) as a function of sampling time, and maximal concentrations measured for each tissue (*C*_max_) and the time of maximal concentrations (*T*_max_) are given. It should be noted that the direct comparison of absolute AUC values for Cbz vs. IR780-oleyl is not the primary purpose, but rather a comparison of the time profiles and relative AUC of the two compounds in different organs. Additionally, the measured concentrations in all organ and serum samples are plotted in Supplementary information Figs. S1 (PEBCA) and S2 (LipImage). Several observations are noteworthy. Most notable is the preferential accumulation of IR780-oleyl in the liver and serum as compared to Cbz. Accumulation in the testes, thymus and brain (both compounds) is limited for all doses. Nevertheless, it is interesting to observe that increase in brain accumulation as a function of dose is much higher for Cbz than for IR780-oleyl. As in the case for serum, IR780-oleyl concentration did not plateau above the baseline which lead to a continuous clear increase in AUC over the analysed 14 days. This was not reflected to the same extent for Cbz.

### Organ accumulation relative to blood is much higher for Cbz than IR780-oleyl

**Fig. 3:**
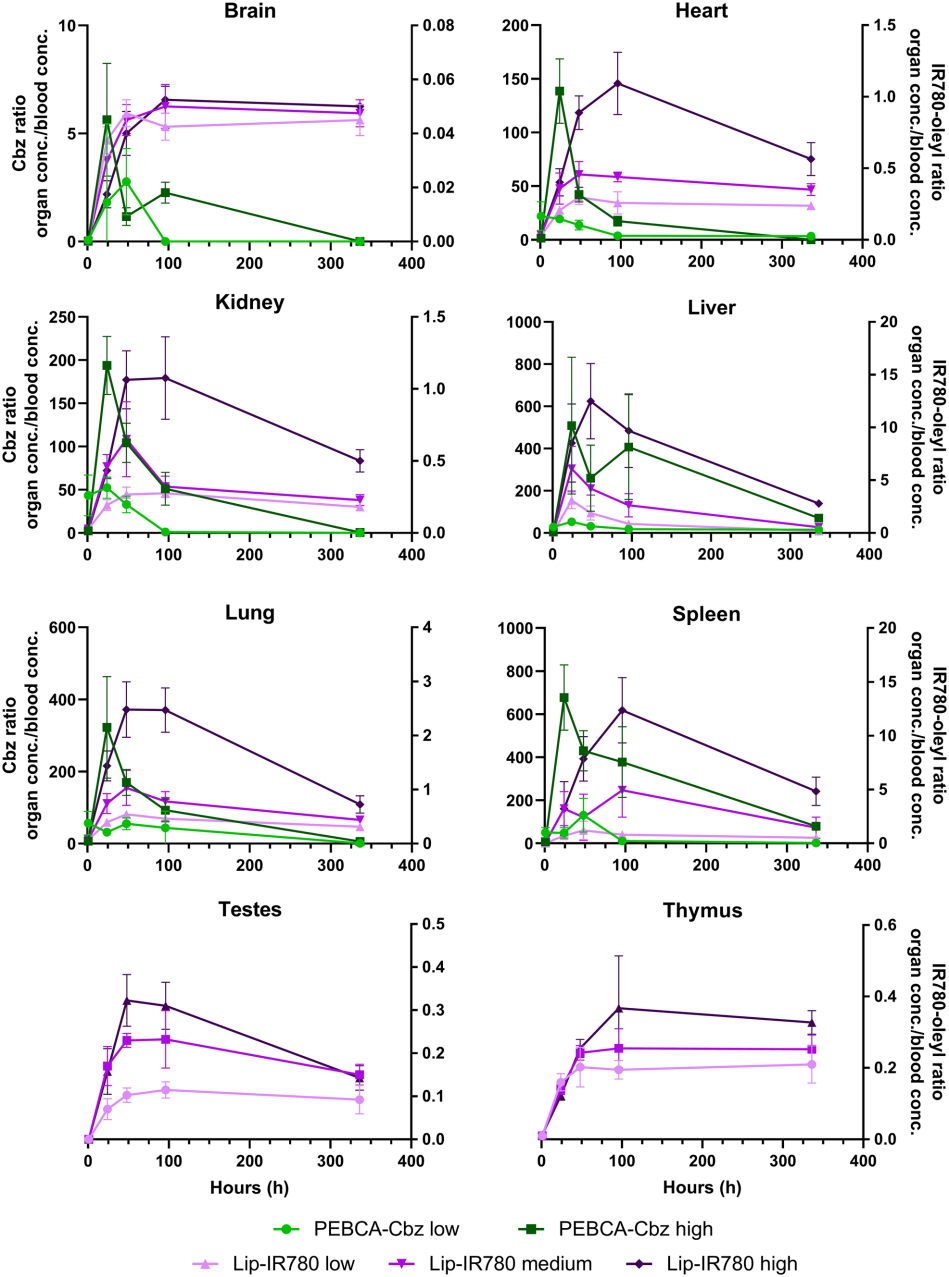
Ratios of measured concentrations (Cbz, IR780-oleyl) (ng/g) in tissue relative to serum at all sampling times and doses. Y axis (scale) for Cbz on the left side; for IR780-oleyl on the right side of each graph. Each data point shows the average and standard deviation of *n* = 4 animals

Compounds—and nanomaterials—in the blood circulation will be in contact with all perfused organs, and relative concentrations will be influenced by both passive, concentration-driven equilibration and physicochemical affinity in the tissue cells and by active transport processes. Therefore, a comparison of the concentration *ratios* of Cbz and IR780-oleyl between the respective organs and the blood could give indications on preferential accumulation. This comparison is shown in Fig. 3 for all organs and doses and for both compounds. Several interesting observations emerge. Most striking is the dramatically higher ratio, i.e. organ accumulation over blood, of Cbz as compared to IR780-oleyl, at peak concentration, of the highest doses. For all tissues except liver, the peak concentration ratio organ:blood is at least 100-fold higher for Cbz than for IR780-oleyl; for liver, it is still about 50-fold higher. The same trends, although slightly less accentuated, are present at lower dose levels. In other words, the enrichment in organs is much higher in the case of Cbz than for IR780-oleyl. Comparing across the dose regimes demonstrates a virtually uniform trend that the concentration ratios organ:blood increase with increasing dose. Finally, it is interesting to observe that for both formulations and all dose regimes, the organ:blood ratio reaches a peak whereafter it decreases, and that this peak is in most cases reached earlier for Cbz (24-h sampling point) than for IR780-oleyl (24- to 96-h sampling point).

### Biodistribution concentrations in tissue are not linear with injected dose

**Fig. 4:**
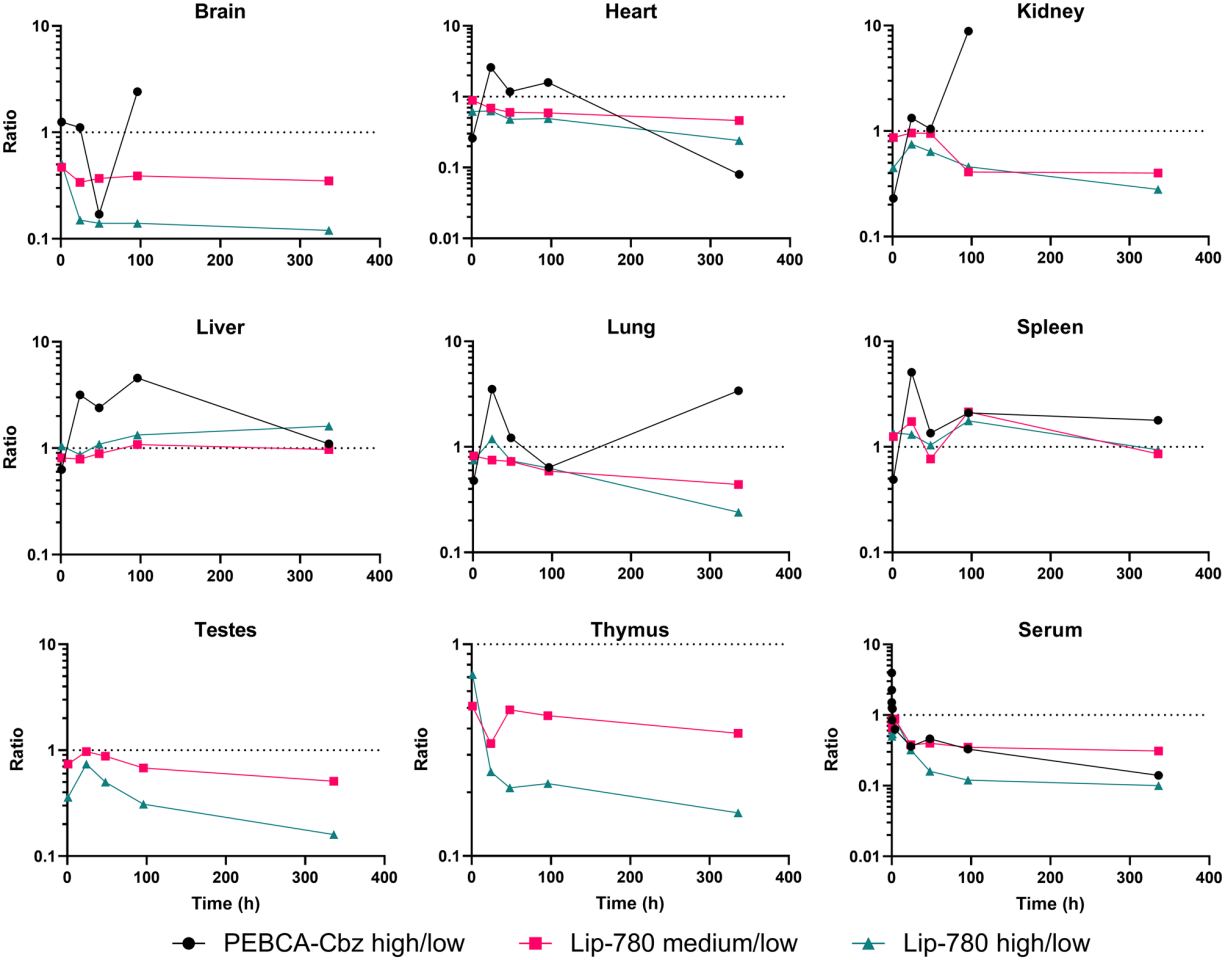
Ratios between analyte concentrations (Cbz after PEBCA injection; IR780-oleyl after LipImage injections) measured in tissues and serum after different injected doses (high/low for PEBCA; high/medium/low for LipImage), at all sampling times. Ratios (logarithmic scale) are normalized to the difference in injected doses, i.e. a ratio = 1 represents perfect linearity in measured vs. injected dose. Note that both axes for the serum data are logarithmic. Each data point shows the average of *n* = 4 animals

As already indicated in Fig. 2, different kinetics are observed for the two PEBCA doses; the relative increase in cumulative AUC over time is clearly higher at the high administered dose, for the heart, kidney, liver, lung and spleen. A similar trend across the three dose levels for LipImage is also observed but differences are less. Figure 4 shows a systematic comparison over the experiment duration of the detected tissue and serum concentrations of Cbz and IR780-oleyl, respectively, plotted as the ratios normalized for differences in administered dose of these markers. A ratio < 1 would correspond to less increase in detected concentration than if the increase was linear as a function of injected dose, which could be perceived as a ‘saturation’ effect. Generally, the deviation from dose linearity over time is similar across the different doses of LipImage, or, as seen for the brain, testes and thymus where detected IR780-oleyl concentrations are low, the deviation from linearity is positively correlated with dose level. For PEBCA, the deviations from dose linearity are generally larger, and with different time profiles; this is particularly visible at the 24-h sampling point and overall in the liver samples, where a majority of the dose response ratios are significantly larger than 1. This reinforces the high degree of organ accumulation observed from Fig. 3.

### Metabolites of Cbz are found primarily in the lung tissue

**Fig. 5:**
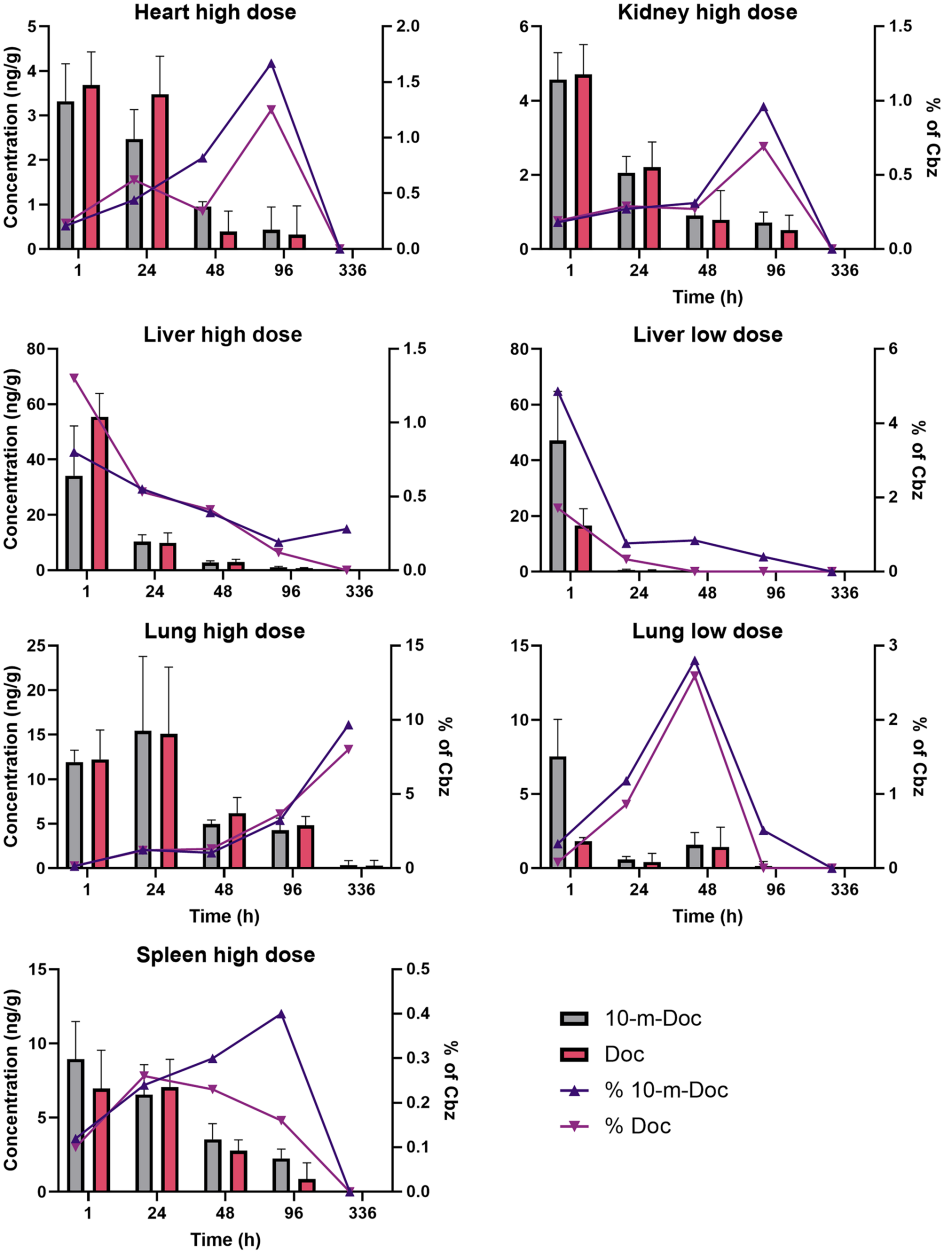
Measured concentrations of the Cbz metabolites 10-methyl-docetaxel (10-m-Doc) and docetaxel (Doc) over all sampling times, after administration of the PEBCA formulation. The graphs include overlay of absolute concentrations (ng/g tissue) given as bars and with y axis on the left side, and as concentrations (%) relative to Cbz in the same samples with y axis on the right side. Each data point shows the average of *n* = 4 animals

Metabolism of Cbz in our experiments occurs first by demethylation at the C7, to form 10-methyl-docetaxel (10-m-Doc), and subsequently demethylation at the C10 position to form docetaxel (Doc). No 7-methyl-docetaxel was observed in any sample, which has been proposed as a possible metabolic pathway, and also no 7-epi-docetaxel. The latter was investigated based on previous observations of the very significant C7 epimerization found to occur in the analogous paclitaxel molecule under physiological conditions. The 10-m-Doc and Doc metabolites are found in low amounts in almost all tissues. Absolute tissue concentrations, as well as the relative amount of each metabolite compared to the parent compound Cbz, are shown in Fig. 5. Concentrations in brain were negligible (0.2 ng/g even at high dose administered) and are therefore not shown. Similarly, only the data from the highest injected dose for the heart, kidney and blood are shown for clarity; concentration trends were similar for lower doses, where metabolites could be detected. In serum, only 10-m-Doc was found, at low concentrations (0.5% or less of Cbz in the same sample), and only up to the 15-min sampling point. For all organs except the liver, there is an initial increase in the proportion of the metabolites with time, even if the absolute amounts go down. Interestingly, for lung at the high dose, there is a trend towards an increase in both absolute and relative concentrations of both metabolites from the 1-h to the 24-h time point, and a further increase in relative amounts throughout the entire experiment.

By far, the highest absolute metabolite concentrations are found at the 1-h sampling point in the liver, for both high and low PEBCA doses, but with a reduction both in absolute and relative concentrations throughout the experiment. It should be noted that the two metabolites, 10-m-Doc and Doc, are similar in concentrations in most cases, except at the 1-h time point in the liver and lung for the low dose of PEBCA, where there is markedly more 10-m-Doc present, and the highest PEBCA dose where the 1-h liver sample clearly shows a higher concentration of Doc. These differences are interesting in that Doc is the metabolic product of 10-m-Doc conversion, in the absence of the 7-methyl-docetaxel pathway.

## Discussion

The animal experiments in the current work were performed at two different partner sites; the LipImage was injected and sampled at RIVM (Netherlands), whereas PEBCA experiments were performed at SINTEF (Norway). This was founded in the aims of the REFINE project, which include interlaboratory comparisons and standardization of procedures and assays. It is well known that animal experiments are logistically very complex and that comparison and replication across studies can be challenging. We have tried to maximize comparability, although the differences in formulation process of the nanoparticles, specifically, challenges in comparatively loading either IR780-oleyl into PEBCA or Cbz into LipImage-type particles, inherently prevent us from obtaining a direct comparison of the nanocarriers as the sole variable. Sampling and processing of blood and tissues was coordinated at the same times. Some additional early blood sampling time points for the PEBCA experiments and two additional organs for the LipImage experiments were added, based on the previous experience with the respective systems. Two dose levels were chosen for the PEBCA experiments, based on the known therapeutic window for animal experiments with Cbz-loaded PEBCA particles. From here on, we will the discuss the findings and compare the two nanoparticles and their biodistribution. But it is of interest to remember that the main goal of the data generated in this study is to feed an in silico biodistribution model generated within the REFINE project with experimental data.

### Analytical method considerations

It should be noted that for both carriers, an encapsulated marker was used to evaluate the tissue distribution using LC–MS/MS requiring destruction of the organs before measurement, so it cannot be determined whether the drug-carrier system was intact or whether the content was released within the evaluated organs. The status of LC–MS/MS as a de facto gold standard for combined biodistribution and pharmacokinetics studies with small molecule drugs leads to some analytical challenges. At the very low instrumental detection limits, adhesion of hydrophobic and charged analytes during sample preparation can severely limit the effective detection limit. The strategy developed in this work builds upon the selectivity and specificity of LC–MS/MS, where it is utilized to quantify low levels of the analytes of interest after addition of high concentrations of Miglyol, which most likely saturates binding sites on labware used that would otherwise lead to loss of target analytes. One could argue that the highly unpolar Miglyol could also contribute to increase effective solubility of the very hydrophobic analytes; this, however, seems speculative considering the very low concentration used, and would need further investigation. We propose that the strategy outlined here could work as an auxiliary sample processing approach for other analytes that exhibit similar challenges with unwanted surface binding.

### Large differences in blood vs. organ accumulation could be related to drug release

One of the most striking results observed in the study was the large difference in concentration ratios between blood (serum) and organs, for the two analytes Cbz and IR780-oleyl. This is clearly visible in the calculated ratios for all samples (Fig. 3). One of the most imminent differences between the LipImage and PEBCA formulations is the particle size, with PEBCA having a particle diameter (121 nm) more than twice that of LipImage (52 nm). Although no clear physiological cut-off is known in this size range, e.g. Li and Huang [36] point to data showing a certain impact of this particle size range on biodistribution of liposomes. Other reviews also clearly indicate an effect of particle size on biodistribution, although the effect is not uniform across nanomaterial types [3, 6]. Surface charge (zeta potential) is relatively similar (and close to neutral) for the two formulations, with PEBCA slightly negative.

The results presented in Fig. 3 are dependent on the method used to calculate the organ/plasma ratio of the analytes. In this case, we have chosen to look at the same time point for both plasma and organs; however, there is a possibility that the nanoparticles and analytes were occupying circulating blood cells (such as macrophages), which could askew the result. IR780-oleyl accumulates to a much lower extent in organs as compared to Cbz. This is exacerbated by the slower elimination of IR780-oleyl from the blood stream (Fig. 1), and the stabilization towards a relatively high (> 25 ng/mL) residual concentration. Besides the marker dose applied, several factors could influence this difference between the two delivery systems, PEBCA and LipImage. Physicochemical aspects such as size, chemical composition of the nanoparticle and surface coverage of PEG are known to influence targeting; e.g. for LNPs, Dilliard et al. investigated the extrahepatic targeting, and proposed a mechanism where shedding of PEG from the particle surface is followed by selective interactions with plasma proteins, based on the chemical identity of the lipids present, and eventually tissue targeting based on the adsorbed proteins [37]. In our work, it could be noted that the length distribution of PEG moieties in the LipImage formulation is slightly shifted towards longer PEG than in the PEBCA formulation. Anselmo et al. [38] showed that for polymeric nanoparticles, material elasticity did influence circulation time and cellular uptake. Another factor that could strongly influence biodistribution of the nanoparticle payload (Cbz, IR780-oleyl) is the extent to which it exists as released, free compound, as opposed to encapsulated in the nanoparticle. Release can, in turn, happen along several routes, either by diffusion from (an intact) particle, by gradual erosion, or by complete dissociation of the particle. Release kinetics of drug molecules from therapeutic nanoparticles can vary dramatically and is influenced by PEGylation and protein corona, especially for hydrophobic drugs and a release diffusion-based release mechanism [39, 40]. Thus, it becomes important to try to quantify which fraction of the payload is released, and which is still encapsulated. At the same time, quantification of the intactness of organic nanoparticles in vivo is very challenging, e.g. due to the presence of high concentrations of plasma proteins that can severely affect light scattering-based particle detection techniques. In a previous animal experiment, an LC–MS/MS method was developed that could quantify single compounds from all the (mono- or polydisperse) constituent lipid classes incorporated in the LipImage formulation, as markers for the nanoparticles, complementary to their IR780-oleyl payload. However, all lipids except the PEGylated Myrj S40 were found to be endogenous in serum and tissues, and no LipImage-related signal beyond background in either serum or the tissues investigated was detected (data not shown). The Myrj S40 signal was not detectable in the serum or tissue samples. For PEBCA, no method currently exists that can track the nanoparticle carrier material with sufficient sensitivity in vivo/ex vivo, implying that quantification of Cbz and IR780-oleyl as proxies for their respective delivery systems is as of yet the best approach. A generic approach quantifying all PEGylated moieties would, however, facilitate detection of virtually all carrier materials currently used; this is currently under investigation in our lab.

Sulheim et al. showed that release rates from other PACA materials (poly(butylcyanoacrylate), PBCA, and poly(octylcyanoacrylate), POCA) were slow for the hydrophobic fluorescent dye NR668 [41] and were dominated by particle degradation rather than diffusion (‘leakage’). Furthermore, the choice of cyanoacrylate monomer was found to impact on the nanoparticle degradation rates, where polymers with shorter alkyl chain lengths degraded faster. Even if it is not obvious that we can extrapolate these results to the PEBCA/Cbz system, the alkyl chain length is intermediate to PBCA and POCA, and Cbz is very hydrophobic, increasing the likelihood that the observed blood and tissue concentrations of Cbz are indeed locally released from PEBCA nanoparticles. For the LipImage, similar studies on particle integrity and drug release in biological matrices are not available. Caputo et al. [42] did measure effect of plasma proteins on particle size distribution (physical stability), but a full kinetic analysis was not performed, and hence, it is not possible to conclude on the particle stability of LipImage in the biological matrices. Jacquart et al. [20] evaluated the fluorescence-based biodistribution and toxicity after intravenous LipImage injection in mice. In the first study, animals were sacrificed at 30 min, 4 h and 24 h; at 4 and 24 h, the fluorescence was highest in the liver. In the second study, animals were sacrificed at 1, 2, 8, 15 and 21 days. A decrease in fluorescence from day 1 was seen in all organs analysed except for the liver that showed a slight increase between day 1 and day 8. In the third study, plasma levels were 7.5%, 2.5% and 0.5% of the injected dose at 30 min, 5 h and 24 h after injection, respectively. Using a mouse tumour model, Genevois et al. [43] showed that 1, 6 and 24 h after intravenous LipImage injection, the fluorescent signal was, next to the subcutaneous tumour, predominantly present in the liver. Ex vivo analysis 24 h after injection showed that the signal was predominantly present in the liver, as well as in the tumour, intestines, kidneys, lung and spleen. Overall, this data is in line with the data obtained in our present study. It should be noted that the dose levels used in the Jacquart et al. study ($\approx$1000 or 2000 mg/kg, depending on the study) were higher than in our study, $\approx$200 mg/kg as highest dose. The very high dose used in the Jacquart et al. study was also confirmed when extrapolating to larger animal models (rat, dog), for which the imaging dose had to be considerably reduced in order not to saturate fluorescence images [15, 21]. In these larger animal models, again similar biodistribution patterns were observed, with fluorescence distribution in all organs at 24 h after injection, though more pronounced in the liver and to a lesser extent in the steroid-rich organs (adrenal, ovaries), intestines, lymph nodes and kidneys. This still does not provide clear information of whether the fluorescent signal arises from free or encapsulated IR780-oleyl. In our experiments, the stable level of IR780-oleyl in the blood for the first hour and its concentration convergence towards approx. 25 ng/mL during the 14 days could conceivably indicate that a reservoir of IR780-oleyl is formed, e.g. in circulating blood cells. Interestingly, Li et al. [44] looked at different nanoformulations of paclitaxel, and found that for all of these, concentrations of the drug were approximately double in whole blood as compared to plasma, indicating that blood cells could act as a reservoir for paclitaxel, conceivably in concentration equilibrium with the free (or protein bound) drug in plasma.

The accumulation of Cbz and IR780-oleyl in the brain was low as the blood–brain barrier protects the brain from foreign substances. However, the uptake of Cbz increased more than expected from the higher dose of PACAB. Compared to other taxanes, such as docetaxel and paclitaxel, Cbz is more hydrophobic and it is hypothesized that it can enter the CNS through cell membrane diffusion [30]. The reduced *C*_max_ seen for Cbz in the brain (Supplementary information Fig. S1) could be explained by Cbz needing to be released from the nanoparticles before it can accumulate in the brain. We have also previously reported uptake of Cbz in the brain from PACA nanoparticles [45]. The delayed peak concentration seen for Cbz was not seen for IR780-oleyl in the brain. However, it can be seen in multiple other organs (Supplementary information Fig. S2), another fact that may point towards a delayed release from a blood reservoir of IR780-oleyl.

Finally, dose linearity (Fig. 4) was limited for almost all organs and formulations tested; only liver concentrations of IR780-oleyl showed an approximately linear transfer over the time course of the experiments. Generally, IR780-oleyl exhibited a dose response < 1 for most organs, suggesting a ‘saturation’ of the tissue (or serum) in question. Interestingly, the trend was opposite for Cbz with a dose response > 1 for a majority of organs and time points, but a dose response < 1 and remarkably similar to that of IR780-oleyl for serum. Overall, it might be argued that this points to a distribution profile that is based on active uptake in organs and blood cells [44], e.g. by the Cbz-containing nanoparticles, rather than an equilibrium driven primarily by a concentration gradient of dissolved Cbz between blood and organs. Another factor that could give similar results would be the saturation of metabolism, yielding and apparent higher concentration of the compound that is metabolized. Interestingly, Shalgunov et al. [46] observed pronounced dose-dependent pharmacokinetics for a model therapeutic nanoformulation based on PLA-PEG, but not for inactive (non-loaded) nanoparticles, indicating that the issue of dose linearity in controlled-release nanoformulations can be quite complex.

### Metabolite analysis yields interesting therapeutic indications

The current work was primarily designed as a comparative biodistribution study, and emphasis has not been on a comprehensive pharmacokinetics study of metabolism. Nevertheless, quantification of two key metabolites from Cbz was performed, showing that demethylation at the C7 position precedes the demethylation at the C10 position. Judging from the similarity in time profile of the two metabolites 10-m-Doc and Doc, it seems that the rate-limiting step is the conversion of Cbz into 10-m-Doc. We also found that C7 epimerization of docetaxel did not occur to any significant extent in these experiments, contrary to what we observed in plasma for the taxane analogue paclitaxel [28]. The high degree of conversion in the liver is expected, based on the presence of cytochrome P450 and other detoxifying enzymes. Nevertheless, the accumulation of the metabolites in lung tissue at late time points is interesting, especially considering that the peak concentration of Cbz (*C*_max_ = 7593 ng/g) is higher than in any other organ, even if it occurs at the earliest sampling point. This could indicate that the nanoformulation used here leads to highly effective delivery to lung tissue and also high degree of tissue retention. No tumour model was used in the current study, but it is recommended to investigate whether an orthotopic lung cancer animal model could benefit from the PEBCA formulation of Cbz used here; conceivably, an even stronger accumulation could be seen in the tumour model due to the EPR effect.

## Conclusions

In the current study, we have shown that two clinically relevant, organic nanoformulations exhibit clear and systematic differences in biodistribution, over time and as function of dose. The observed differences indicate that for different nanocarrier formulations, each formulation needs to be evaluated independently, and that possibilities for read across may be limited. One crucial element that could not be fully elucidated was the relative proportions of released vs. encapsulated nanoparticle payloads (IR780-oleyl, Cbz). This is critical to determine for bioactive compounds like cabazitaxel, as only the free drug will exert a pharmacological effect. Methods to independently determine, in vivo or ex vivo, concentrations of both the payload and the nanoparticles are needed, in order to support progression and clinical translation of promising nanomedicines and nanomedical devices, as was already identified as critical methodological gaps in the assessment of nanobiomaterials [7].

We found notable differences in biodistribution of the formulations studied. The most striking was the apparent preferential accumulation of Cbz in organs; the concentration ratio organ:blood was orders of magnitude higher than what was seen for IR780-oleyl. It should be noted that this observation could also be related to metabolic conversion, which was observed and quantified for Cbz, whereas the metabolism of IR780-oleyl could not be elucidated. Interestingly, Cbz, and its metabolites, showed a notable and prolonged accumulation in lung tissue compared to other organs. Clearly, more detailed ADME studies are needed, but this could support the investigation of Cbz as a therapeutic option for lung cancer treatment, which is currently not indicated. Furthermore, it could indicate an advantage from nanoencapsulation of Cbz as compared to the free drug, for which off-target toxicity is currently dose-limiting.

## Supplementary Information

Below is the link to the electronic supplementary material.

Supplementary file1 (DOCX 137 KB)

## Data Availability

The datasets generated during and/or analysed during the current study are available from the corresponding author on reasonable request.
