# Supplementary material for: A comparative biodistribution study of polymeric and lipid-based nanoparticles
Source: Drug Deliv Transl Res. 2022 Apr 15;12(9):2114–31. doi: 10.1007/s13346-022-01157-y (PMC9012159; doi:10.1007/s13346-022-01157-y)
Supplement: Supplementary file 1 — Supplementary file1 (DOCX 137 KB) [file 13346_2022_1157_MOESM1_ESM.docx]

# Supplementary data

Figure S1: Concentration of Cbz in organs over time. Light green circles is PEBCA low dose and dark green is PEBCA high dose.

Figure S2: Concentration of IR780-oleyl in organs over time. From lighter to darker purple: LipImage low dose (circle), LipImage medium dose (square), LipImage high dose (triangle).
